# Primary results from EL1SSAR, a prospective phase IIIb study of first-line atezolizumab plus nab-paclitaxel therapy for patients with PD-L1-positive advanced triple-negative breast cancer

**DOI:** 10.1016/j.breast.2026.104836

**Published:** 2026-06-08

**Authors:** L. Gianni, S. Delaloge, E. Ciruelos, O. Trédan, C. Castañeda, M.H. Abreu, I. Paris, M. Colleoni, G. Scandurra, M. Kwiatkowski, N. Antone, V. Gregorc, P. Tagliaferri, S. Borštnar, I. Blancas, M. Raimondo, M. Alfie, C. Arce-Salinas, P.S. Madeira, Y.G. Lin, C. Ye, K. Koli, J. Mouta, M. Martin

**Affiliations:** a Fondazione Michelangelo, Milan, Italy; b Department of Cancer Medicine, Gustave Roussy, Villejuif, France; c Hospital Universitario 12 de Octubre, Madrid, Spain; d Department of Medical Oncology, Centre Léon Bérard, Lyon, France; e Instituto Nacional de Enfermedades Neoplásicas, Lima, Peru; f Medical Oncology Department, Portuguese Institute of Oncology, Porto, Portugal; g Gynecologic Oncology Unit, Department of Women and Child Health, Fondazione Policlinico Universitario A. Gemelli, IRCCS, Rome, Italy; h Division of Medical Senology, Istituto Europeo di Oncologia (IEO) IRCCS, Milan, Italy; i Medical Oncology Ospedale Cannizzaro, Catania, Italy; j University of Enna, Enna, Italy; k Szpital Wojewódzki Im. Mikołaja Kopernika, Koszalin, Poland; l The “Prof. Dr. Ion Chiricuta” Institute of Oncology, Cluj-Napoca, Romania; m Clinical Research and Innovation, Candiolo Cancer Institute, FPO-IRCCS, Candiolo (TO), Italy; n Azienda Ospedaliera Universitaria “Renato Dulbecco” and DMSC Magna Grecia University, Catanzaro, Italy; o Institute of Oncology, Ljubljana, Slovenia; p Faculty of Medicine, University of Ljubljana, Ljubljana, Slovenia; q Hospital Universitario San Cecilio, Granada, Spain; r Departamento de Medicina, Granada University, Granada, Spain; s Instituto de Investigación Biosanitaria (IBS), Granada, Spain; t Sanatorio de La Mujer, Rosario, Argentina; u Organizacion Medica de Investigacion, Buenos Aires, Argentina; v Instituto Nacional de Cancerologia, Departamento de Tumores Mamarios/Oncología Médica, Ciudad de México, Mexico; w Servico de Oncologia Médica, Instituto Português de Oncologia de Coimbra Francisco Gentil, Coimbra, Portugal; x Product Development Oncology, Genentech, South San Francisco, CA, USA; y Biostatistics, Genentech, South San Francisco, CA, USA; z Product Development Safety, Genentech, South San Francisco, CA, USA; aa Global Product Development Medical Affairs Oncology, Roche Farmacêutica Química, Lda, Amadora, Portugal; ab Hospital General Universitario Gregorio Marañón, Universidad Complutense, Madrid, Spain

**Keywords:** PD-L1, Immune checkpoint inhibitor, Triple-negative breast cancer, SP142, ECOG

## Abstract

**Background:**

IMpassion130 trial results led to approval of first-line atezolizumab plus nab-paclitaxel for PD-L1-positive advanced triple-negative breast cancer (aTNBC). The global single-arm EL1SSAR study (NCT04148911) enrolled a broader patient population to elucidate outcomes in routine practice.

**Methods:**

Patients with measurable PD-L1-positive aTNBC and no prior systemic therapy for advanced disease received atezolizumab (840 mg, days 1&15) plus nab-paclitaxel (100 mg/m^2^, days 1,8,15) every 28 days until disease progression or unacceptable toxicity. PD-L1 status was assessed locally (mandatory) and centrally (optional) using the VENTANA PD-L1 (SP142) Assay. Patients with stable asymptomatic CNS metastases, ECOG PS 2 or selected autoimmune diseases were allowed. Primary endpoints were the incidence of grade ≥2 immune-mediated and grade ≥3 adverse events (AEs). Secondary endpoints included overall survival and investigator-assessed progression-free survival (PFS). PD-L1 testing concordance was an exploratory endpoint.

**Results:**

Among 182 treated patients, five had CNS metastases and three had ECOG PS ≥ 2. Patients received a median of six treatment cycles (range 1–63); 7% were treated for >3 years. Grade ≥2 immune-mediated AEs occurred in 12% of patients (95% CI 8%–18%) and grade ≥3 AEs in 47% (95% CI 39%–54%). There were no treatment-related deaths. Median PFS was 7.4 (95% CI 5.6–10.6) months (>2 years in 27 patients [15%]) and median overall survival was 27.0 (95% CI 22.0–33.8) months. Concordance between local and central PD-L1 testing was 67% (65/97). Median PFS was >11 months in patients with centrally confirmed PD-L1-positive status.

**Conclusions:**

Safety and efficacy were consistent with results from IMpassion130, which enrolled a more selected population.

## Highlights

• The single-arm EL1SSAR study evaluated first-line atezolizumab + nab-paclitaxel in 182 patients with PD-L1+ advanced TNBC.
• Eligibility criteria were chosen to enrol a patient population representative of real-world practice.
• Safety (primary endpoint) and efficacy were consistent with results from the IMpassion130 randomised phase III trial.
• Treatment was continued for >3 years in 7% of patients.
• Median PFS was >11 months in patients with centrally confirmed PD-L1-positive status.

## 1 Introduction

The integration of immune checkpoint inhibitors into standard therapy has transformed outcomes for patients with triple-negative breast cancer (TNBC) [1,2]. The combination of atezolizumab and nab-paclitaxel is an approved first-line regimen for PD-L1-positive locally advanced or metastatic TNBC based on positive results from the randomised phase III IMpassion130 trial [3,4], and is recommended in international guidelines [[5], [6], [7]]. While prospective, double-blind, placebo-controlled randomised phase III trials are the gold standard for establishing efficacy and safety, they do not always answer important practical clinical questions, including how outcomes observed in registrational pivotal trials translate for populations presenting in everyday oncology practice [8]. Patient populations typical of routine practice – for example, patients with Eastern Cooperative Oncology Group performance status (ECOG PS) ≥2 – may be under-represented or excluded from pivotal trials [9,10]. The IMpassion130 trial also excluded patients with untreated central nervous system (CNS) disease (although patients with asymptomatic treated CNS metastases were permitted) and a history of autoimmune disease or previous immune checkpoint-targeting therapies [3]. To gain a better understanding of outcomes and tolerability in these understudied populations and to address some of the knowledge gaps surrounding immune checkpoint inhibitors, the single-arm EL1SSAR study enrolled a broader population of patients to elucidate safety and efficacy in settings more representative of routine clinical practice.

## 2 Methods

The international, open-label, single-arm phase IIIb EL1SSAR study (NCT04148911) was conducted in Italy, Spain, Portugal, Poland, Romania, Peru, France, Hungary, Argentina, Slovenia, Mexico, the Czech Republic and Chile. It was conducted in full conformance with the International Council for Harmonisation E6 guideline for Good Clinical Practice and the principles of the Declaration of Helsinki or applicable laws and regulations of each participating country, whichever afforded greater protection to the individual. The study complied with the requirements of the International Council for Harmonisation E2A guideline (Clinical Safety Data Management: Definitions and Standards for Expedited Reporting), the EU Clinical Trials Directive (2001/20/EC) or Clinical Trials Regulation (536/2014) and applicable local, regional and national laws.

Patients with measurable unresectable locally advanced or metastatic TNBC (aTNBC) who had received no prior systemic therapy for aTNBC were screened for eligibility. Taxane therapy for early-stage disease was permitted providing the treatment-free interval was ≥12 months. Non-taxane chemotherapy agents for early-stage disease (including capecitabine) were permitted if the treatment-free interval was ≥6 months, broadening the population enrolled in IMpassion130 to reflect the widespread use of capecitabine in patients with residual disease following neoadjuvant chemotherapy. Patients with ECOG PS 2 or selected autoimmune diseases (see Appendix A) were eligible, as were patients with stable asymptomatic CNS metastases (providing they were limited to the supratentorial region or cerebellum and did not require ongoing corticosteroid therapy for CNS disease). Patients had to be aged ≥18 years with adequate haematological, renal and hepatic function; written informed consent was required from all patients.

TNBC (negative HER2, oestrogen receptor and progesterone receptor status) was determined according to American Society of Clinical Oncology/College of American Pathologists guidelines [[11], [12], [13]], assessed locally. PD-L1 status was assessed locally to determine eligibility using the VENTANA PD-L1 (SP142) Assay (Roche Diagnostics, Rotkreuz, Switzerland). Patients with PD-L1-positive tumours, defined as PD-L1 expression on ≥1% of tumour-infiltrating immune cells, were enrolled and treated with atezolizumab (840 mg on days 1 and 15) plus nab-paclitaxel (100 mg/m^2^ on days 1, 8 and 15), repeated every 28 days until disease progression, unacceptable toxicity, loss of clinical benefit in the opinion of the investigator or withdrawal of consent. Central testing of PD-L1 status using the VENTANA PD-L1 (SP142) Assay was optional.

Adverse events (AEs) were assessed at regular intervals, graded according to the National Cancer Institute Common Terminology Criteria for Adverse Events version 5.0 until 30 days (90 days for serious AEs and AEs of special interest) after the last dose of study treatment, or the start of new anti-cancer therapy if earlier. Disease was assessed every 8 weeks during the first year and then at least every 6 months until disease progression, death or consent withdrawal. Follow-up for subsequent anti-cancer therapy and survival was continued for up to 3 years after the last patient was enrolled.

Primary endpoints were the incidence of grade ≥3 AEs and the incidence of grade ≥2 immune-mediated AEs, defined as AEs of special interest (according to pre-defined medical concept categories related to the drug mechanism of action) that were ongoing on initiation of systemic corticosteroid therapy and for which systemic corticosteroid therapy was administered ≤30 days after the AE began. Secondary endpoints included the incidence of AEs of any grade, the incidence of serious AEs and efficacy endpoints including overall survival (OS) and investigator-assessed progression-free survival (PFS) according to Response Evaluation Criteria in Solid Tumours (RECIST) version 1.1. OS and PFS were analysed in all patients and in the subgroup of patients with centrally confirmed PD-L1-positive status. Exploratory endpoints included the investigator-assessed objective response rate according to RECIST version 1.1, disease control rate (complete or partial response or stable disease, regardless of duration), duration of response in responding patients, confirmed objective response rate and duration of confirmed response based on two consecutive investigator assessments, PD-L1 testing concordance between local and central PD-L1 assessment, and safety and efficacy in clinically relevant subgroups (patients with CNS metastases, ECOG PS 2, prior anti-cancer treatment, prior PD-[L]1 inhibitor therapy, and taxane versus non-taxane [neo]adjuvant therapy). In a post hoc analysis, outcomes were also evaluated in the subgroup of patients whose tumours were PD-L1-positive by local assessment (for eligibility) but PD-L1-negative by central assessment.

It was planned to enrol approximately 180 patients. Assuming that approximately 40% of screened patients would have PD-L1-positive tumours, it was anticipated that approximately 450 patients would have to be screened to enrol 180 patients with PD-L1-positive aTNBC. The final analysis was planned after all patients had died, withdrawn consent, been lost to follow-up or had been followed for >3 years after enrolment of the last patient, whichever occurred first. All analyses were performed on the safety population comprising all enrolled patients who received at least one dose of study drug. The primary safety variables are presented as descriptive statistics with corresponding 95% Clopper-Pearson confidence intervals (CIs). The precision of rare AE incidence estimates is presented by two-sided exact 95% Clopper-Pearson CIs in Appendix B. Kaplan–Meier methodology was used to estimate OS and PFS, including 1-, 2-, and 3-year OS and 1-year PFS rates.

## 3 Results

### 3.1 Patient population

Between 10 December 2019 and 15 December 2021, 182 female patients were enrolled from sites in 13 countries across Europe and North and South America (Appendix C). All 182 patients received at least one dose of study therapy, representing the safety population. Five patients had known CNS metastases, three had ECOG PS ≥ 2, 25% had de novo metastatic TNBC, 62% had invasive breast cancer of no special type (previously known as ductal histology) and 58% had previously received (neo)adjuvant systemic therapy, most commonly anthracycline- and/or taxane-containing (Table 1). None of the patients had previously received an anti-PD-(L)1 therapy. Most patients (82%) had ongoing comorbidities at baseline, including hypertension in 27%, hypothyroidism in 14%, anaemia in 10% and insomnia and hypercholesterolaemia each in 8%. Fourteen patients (8%) had permitted autoimmune diseases as detailed in Appendix A, of whom 11 (6%) had active autoimmune disease at study entry.

**Table 1:** Baseline characteristics.

Characteristic, *n* (%)	All patients (*n = *182)
Age, years	
Median (range)	54 (27–83)
18–40	23 (13)
41–64	118 (65)
≥65	41 (23)
Sex	
Female	182 (100)
Race	
American Indian/Alaska Native	17 (9)
Asian	1 (1)
Black/African American	1 (1)
White	146 (80)
Unknown	17 (9)
ECOG PS	
0	127 (70)
1	51 (28)
≥2	3 (2)
Missing	1 (1)
Pre-existing autoimmune disease	14 (8)^a^
Active at study entry	11 (6)
Histology^b^	
Invasive breast cancer of no special type	113 (62)
Lobular	9 (5)
Medullary	5 (3)
Tubular	1 (1)
Comedo	1 (1)
Other	18 (10)
Not otherwise specified	45 (25)
Grade	
Well differentiated	16 (9)
Moderately differentiated	25 (14)
Poorly differentiated	95 (52)
Unknown	46 (25)
Disease status	
Locally advanced unresectable	28 (15)
Metastatic	154 (85)
De novo metastatic	46 (25)
Metastatic sites	
Liver	33 (18)
Lung	80 (44)
Lymph node status	
Positive	74 (41)
Negative	89 (49)
Missing	19 (10)
Prior anti-cancer therapy	
Radiotherapy	87 (48)
Systemic therapy	106 (58)
Neoadjuvant	56 (31)
Adjuvant	77 (42)
Taxane	92 (51)
PD-(L)1 inhibitor	0

ECOG PS, Eastern Cooperative Oncology Group performance status.

a Hashimoto disease (*n* = 4, all ongoing at study start), rheumatoid arthritis (*n* = 4, one resolved and three ongoing at study start), autoimmune-mediated hypothyroidism (*n* = 2, both ongoing at study start), psoriasis (*n* = 2, both ongoing at study start, one of them concurrently with autoimmune-mediated hypothyroidism above), dermatomyositis (*n* = 1, resolved before study start), lupus erythematosus (*n* = 1, ongoing at study start), Sjogren syndrome (*n* = 1, resolved before study start).

b More than 1 answer possible.

### 3.2 Treatment exposure

At the data cut-off for the final analysis (4 February 2025), the median duration of follow-up was 22.2 months (interquartile range 9.5–39.6 months). All patients had discontinued treatment. Patients received a median of six cycles of atezolizumab (range 1–62) and six cycles of nab-paclitaxel (range 1–63). In 12 patients (7%), treatment was continued for >3 years (both agents for >3 years in eight patients, atezolizumab for >3 years and nab-paclitaxel for <3 years [range 5–24 months] in three patients, and nab-paclitaxel for >3 years and atezolizumab for <3 years [35 months] in one patient). The most common reason for discontinuing either treatment was disease progression (atezolizumab 65%, nab-paclitaxel 54%). AEs led to the discontinuation of nab-paclitaxel in 21% of patients and discontinuation of atezolizumab in 9%. At least one nab-paclitaxel dose modification was implemented because of AEs in 15% of patients.

### 3.3 Safety

Grade ≥3 AEs occurred in 85 patients (47%; 95% CI 39% to 54%) (Table 2). The most common grade ≥3 AE was neutropenia (10%), followed by asthenia, paraesthesia, neutrophil count decreased, white blood cell count decreased (each in 4%), anaemia, leukopenia and alanine aminotransferase (ALT) increased (each in 3%) (Table 3). AEs were grade 4 in 6% of patients and grade 5 in 2%, but there were no treatment-related grade 5 AEs.

**Table 2:** Summary of safety, including primary endpoints (*n = *182).

AE	No. of patients (%)
Any AE	174 (96)
Treatment-related AE	153 (84)
Related to nab-paclitaxel	151 (83)
Related to atezolizumab	113 (62)
Grade ≥3 AE	85 (47)
Grade 5 AE	3 (2)^a^
Serious AE	30 (16)
Treatment-related serious AE	12 (7)
AE of special interest	112 (62)
Grade ≥2 immune-mediated AE	22 (12)
AE leading to any study drug discontinuation	41 (23)
AE leading to atezolizumab discontinuation	16 (9)^b^
AE leading to any study drug interruption or dose modification	108 (59)
AE leading to atezolizumab interruption	97 (53)

AE, adverse event.

a One case each of pneumonitis, pulmonary oedema and cardiorespiratory arrest, all considered unrelated to study treatment.

b One case each of renal failure and hypokalaemia (in the same patient), hypercreatininaemia and peripheral neuropathy (in the same patient), hepatic failure, hepatitis, immune-mediated hepatitis, gamma-glutamyl transferase increased, lipase increased, transaminases increased, arthralgia, myositis, peripheral sensory neuropathy, gastritis, myelitis, chronic myelomonocytic leukaemia, acute interstitial pneumonitis, thrombophlebitis.

**Table 3:** Most common AEs (>10% of patients at any grade or >2% of patients at grade ≥3) (*n = *182).

	No. of patients (%)				
AE	Any grade	Grade ≥3	Grade 3	Grade 4	Grade 5
Any	174 (96)	85 (47)	71 (39)	11 (6)	3 (2)
Alopecia	72 (40)	NA	NA	NA	NA
Anaemia	68 (37)	6 (3)	6 (3)	0	0
Asthenia	61 (34)	7 (4)	6 (3)	1 (1)	0
Nausea	50 (27)	1 (1)	1 (1)	0	0
Diarrhoea	49 (27)	2 (1)	2 (1)	0	0
Neutropenia	44 (24)	18 (10)	12 (7)	6 (3)	0
Fatigue	35 (19)	3 (2)	3 (2)	0	0
Constipation	34 (19)	0	0	0	0
Hypothyroidism	34 (19)	0	0	0	0
Pyrexia	32 (18)	1 (1)	1 (1)	0	0
Arthralgia	30 (16)	0	0	0	0
Myalgia	30 (16)	0	0	0	0
ALT increased	29 (16)	6 (3)	6 (3)	0	0
COVID-19	28 (15)	0	0	0	0
Leukopenia	27 (15)	6 (3)	6 (3)	0	0
Paraesthesia	27 (15)	7 (4)	7 (4)	0	0
AST increased	24 (13)	4 (2)	4 (2)	0	0
Headache	24 (13)	0	0	0	0
Peripheral neuropathy	24 (13)	5 (3)	5 (3)	0	0
Rash	22 (12)	0	0	0	0
Vomiting	22 (12)	0	0	0	0
Back pain	19 (10)	0	0	0	0
Cough	19 (10)	1 (1)	1 (1)	0	0
WBC count decreased	19 (10)	7 (4)	7 (4)	0	0
Peripheral oedema	16 (9)	4 (2)	4 (2)	0	0
Neutrophil count decreased	15 (8)	7 (4)	7 (4)	0	0
Neurotoxicity	8 (4)	5 (3)	5 (3)	0	0

AE, adverse event; ALT, alanine aminotransferase; AST, aspartate aminotransferase; NA, not applicable; WBC, white blood cell.

**Fig. 1:**
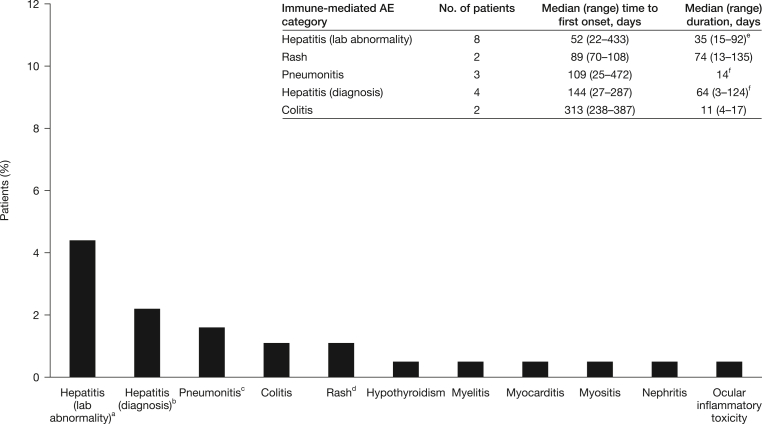
**Incidence, time to onset and duration of grade ≥2 immune-mediated AEs.** The inset table includes grade ≥2 immune-mediated AEs occurring in >1 patient. If a patient had >1 episode of an immune-mediated AE within a single category, median time to first onset was calculated based on the earliest immune-mediated AE and median duration was calculated based on the longest lasting. AE, adverse event; ALT, alanine aminotransferase; AST, aspartate aminotransferase. ^a^Including ALT increased, AST increased, transaminases increased, blood bilirubin increased, gamma-glutamyl transferase increased and hypertransaminasaemia. ^b^Hepatic cytolysis, hepatic failure, hepatitis and immune-mediated hepatitis. ^c^Including acute interstitial pneumonitis. ^d^Maculopapular rash and pustular rash. ^e^Missing in 1 patient. ^f^Missing in 2 patients.

Grade ≥2 immune-mediated AEs occurred in 22 patients (12%; 95% CI 8% to 18%), the most common being hepatitis as a laboratory abnormality (ALT, aspartate aminotransferase, transaminases, blood bilirubin or gamma-glutamyl transferase increased, or hypertransaminasaemia) (Fig. 1 and Supplementary Table A1). Hepatitis as a laboratory abnormality typically emerged within the first 2 months of treatment and the median duration was 1.1 months (Fig. 1). Clinically diagnosed hepatitis was less common, tended to occur later (median time to first onset: 4.7 months) and lasted for a median of 2.1 months. Grade ≥2 immune-mediated pneumonitis was infrequent (occurring in three patients) and showed no clear pattern in time to onset (emerging 0.8, 3.6 and 15.5 months after the first dose). There was one fatal case of pneumonitis (on day 109 in a 79-year-old patient) and one grade 4 immune-mediated AE (transaminases increased, emerging on day 43 and lasting for 92 days). Neither of these AEs was considered by the investigator to be related to study treatment.

All but eight patients (174; 96%; 95% CI 92% to 98%) experienced at least one AE of any grade. The most common any-grade AEs were alopecia, anaemia, asthenia, nausea, diarrhoea and neutropenia (Table 3). AEs were classified as serious in 30 patients (16%; 95% CI 11% to 23%), but serious AEs were considered related to treatment in only 12 patients (7%) (Table 2).

### 3.4 Efficacy

**Fig. 2:**
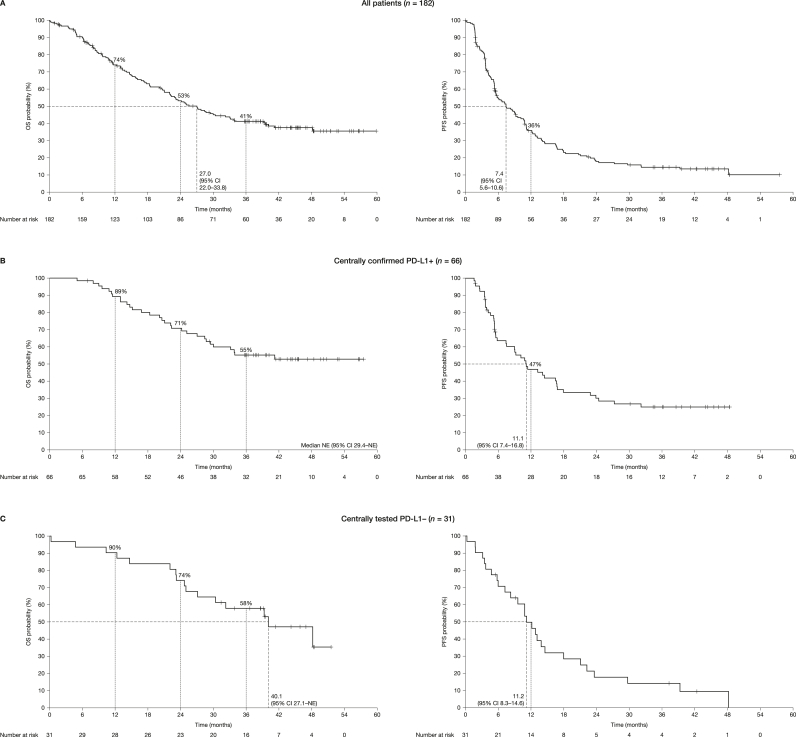
OS and PFS overall and by centrally tested PD-L1 status. A) All patients (*n = *182; pre-specified secondary endpoint). B) Centrally confirmed PD-L1+ (*n = *66; pre-specified secondary endpoint). C) Discordant PD-L1 status (centrally PD-L1–, locally PD-L1+; *n = *31; post hoc analysis) OS, overall survival; PFS, progression-free survival.

By the data cut-off date for this final analysis, 145 of 182 treated patients (80%) had experienced a PFS event and 104 patients (57%) had died (94 [52%] from disease progression, three [2%] from AEs [all considered unrelated to treatment: pulmonary oedema 12 days after last dose, cardiorespiratory arrest 20 days after last dose, pneumonitis 35 days after last dose] and seven [4%] from other causes). Median OS was 27.0 months (95% CI 22.0–33.8 months). OS rates at 1, 2 and 3 years were 74% (95% CI 68% to 81%), 53% (95% CI 46% to 61%) and 41% (95% CI 34% to 49%), respectively (Fig. 2A). Median PFS was 7.4 months (95% CI 5.6–10.6 months) and the 1-year PFS rate was 36%. Of note, PFS exceeded 2 years in 27 patients (15%). Compared with the overall population, these 27 patients with sustained disease control appeared to be more likely to have ECOG PS 0, invasive breast cancer of no special type, locally advanced unresectable or de novo metastatic disease, and less likely to have liver metastases at enrolment (Supplementary Table A2).

In the subgroup of 66 patients with centrally confirmed PD-L1-positive aTNBC, median OS was not estimable (NE; 95% CI 29.4 months–NE) with events in only 30 patients (45%) (Fig. 2B). Median PFS was 11.1 months (95% CI 7.4–16.8 months) and 18 patients (27%) had PFS of >2 years. PFS and OS in additional pre-specified subgroups are shown in Supplementary Table A3.

The confirmed objective response rate was 44% (95% CI 37% to 51%), including complete responses in 12% of patients. The median duration of response in the 80 patients with a confirmed response was 15.0 months (95% CI 10.5–21.2 months). The unconfirmed objective response rate was 55% (95% CI 47% to 62%), including complete responses in 14%. The median duration of unconfirmed response was 9.6 months (95% CI 7.5–15.0 months). A further 28% of patients had stable disease, giving a disease control rate of 82% (95% CI 76% to 88%).

### 3.5 PD-L1 testing concordance

PD-L1 was tested on metastatic samples in 35 of 182 patients (19%; most commonly lymph nodes and lung lesions) and locoregional samples in 121 patients (66%). Optional central PD-L1 testing was undertaken in samples from 97 patients (53% of treated patients). Of these, 65 samples were PD-L1 positive by both local and central testing, representing 67% concordance, 31 were PD-L1 negative by central testing and one was PD-L1 positive by central testing but recorded as PD-L1 0 by local assessment (Supplementary Table A4). To understand the potential clinical and practical implications of this discordance, efficacy was explored in post hoc analyses of the 31 patients with PD-L1-negative status on central testing (despite PD-L1-positive status on local testing). Median PFS was 11.2 months (95% CI 8.3–14.6 months), 16% had PFS >2 years and median OS was 40.1 months (95% CI 27.1 months–NE) (Fig. 2C). OS rates at 1, 2 and 3 years were 90%, 74% and 58%, respectively.

### 3.6 Subsequent therapy

Overall, 109 patients (60%) received subsequent therapy, most commonly including platinum (33%) or capecitabine (31%) (Supplementary Table A5). Antibody–drug conjugates were administered in 32 patients (18%; sacituzumab govitecan in 30 patients, trastuzumab deruxtecan in three patients [both in one patient]). Among the 73 patients who had not received subsequent therapy by the data cut-off, 35 had died, 11 were still on study treatment, 10 discontinued from the study within 90 days of their last dose of study therapy and 17 discontinued from the study >90 days after their last dose (reason for study discontinuation: study termination [*n* = 11; 10 with ongoing response], patient withdrawal [*n* = 5], lost to follow-up [*n* = 1]) without any record of further therapy.

## 4 Discussion

The global single-arm EL1SSAR study was designed to evaluate outcomes with first-line atezolizumab plus nab-paclitaxel in a broader population of patients with PD-L1-positive aTNBC, including understudied populations typically excluded from randomised trials. The safety profile of the regimen was consistent with the known safety profiles of the study drugs and the IMpassion130 results [3] and there were no new safety findings. There was a relatively low incidence of grade ≥2 immune-mediated AEs (12%) and no treatment-related deaths. Deeper analysis of grade ≥2 immune-mediated AEs (primary endpoint) revealed that hepatic laboratory abnormalities occurred early in treatment but few patients developed clinically diagnosed hepatitis, which typically emerged later and persisted for longer. There was no clear pattern in the time to onset of grade ≥2 immune-mediated pneumonitis. However, with so few cases it is difficult to draw conclusions from these findings. In a larger analysis pooling safety data from studies with atezolizumab monotherapy, the median time to onset of pneumonitis was 3.4 months [Roche data on file]. Clinicians should be vigilant for signs of pneumonitis, as it can occur soon after initiating treatment or occasionally later in the course of treatment. Grade ≥3 AEs, which were reported in 47% of patients, were predominantly AEs associated with the chemotherapy backbone (haematological, paraesthesia, neuropathy) and of grade 3 intensity.

AEs leading to interruption of atezolizumab were slightly more common in EL1SSAR than in the IMpassion130 trial (53% versus 31%, respectively); however, AEs related to study treatment were less common (84% versus 96%). These subtle differences may reflect increased familiarity with the safety profile of atezolizumab as this therapy has become established in clinical practice and recognition and management of AEs has improved with experience and education. Other possible explanations include differences in the racial composition and geographical footprint of the study populations, which may affect tolerability [14,15], or differences in other patient characteristics. In this global study aiming to enrol a broader population, most patients (80%) were White and the baseline characteristics of the EL1SSAR patient population were similar to those of the IMpassion130 PD-L1-positive population treated with atezolizumab and nab-paclitaxel. The most notable differences were the higher proportion of patients with ECOG PS 0 (70% in EL1SSAR despite the broadened eligibility criteria for ECOG PS versus 58% in IMpassion130), the lower proportion with liver metastases (18% versus 24%, respectively) and the lower proportion with prior systemic anti-cancer therapy (58% versus 68%, respectively) and prior radiotherapy (48% versus 64%, respectively) [3,4].

PFS and OS outcomes in the overall population of EL1SSAR (PD-L1-positive by local assessment) were in line with efficacy in the PD-L1-positive (by central assessment) population of the IMpassion130 trial (median PFS: 7.4 versus 7.5 months, respectively; median OS: 27.0 versus 25.4 months, respectively; 2-year OS rate: 53% versus 54%, respectively) [3,4]. Results were also consistent with PFS and OS results reported for the combination of pembrolizumab and chemotherapy in the PD-L1-positive populations (combined positive score >10) of the KEYNOTE-355 [16,17] and ASCENT-04/KEYNOTE-D19 [18] trials, all in patients with no previous exposure to an immune checkpoint inhibitor. However, differences in the duration of follow-up and assessment schedules as well as study designs and conduct should be considered when comparing EL1SSAR with these datasets. In EL1SSAR, outcomes appeared to be more favourable in patients with less prior therapy (any anti-cancer therapy, taxane therapy), although results in small subgroups should be interpreted cautiously.

Despite the superficially similar outcomes, closer examination of PFS and OS results reveals intriguing nuances. Central testing of PD-L1 status in EL1SSAR was optional and was undertaken in only half of the patients. Interestingly, there was relatively poor concordance (67%) between local and central PD-L1 testing (although a complete view of assay agreement is not possible in a study limiting enrolment to patients identified as having positive PD-L1 status). Similar findings have been reported from the VANESSA study exploring PD-L1 testing in a global population [19]. Among the 66 patients with concordant locally and centrally determined PD-L1-positive status, median PFS was 11.1 months (95% CI 7.4–16.8 months) and median OS was NE (95% CI 29.4 months–NE). One hypothesis for the striking outcomes in this subgroup was that central PD-L1 testing was more reliable than local testing for identifying patients most likely to have the best clinical outcomes on first-line atezolizumab and nab-paclitaxel combination therapy. However, post hoc analyses of outcomes in the 31 patients with centrally determined PD-L1-negative samples showed that median PFS and OS were at least as favourable as outcomes in the subgroup with centrally tested PD-L1-positive samples. This suggests that the remaining subgroup of the overall population (i.e., those whose samples were not centrally tested) had notably poorer outcomes and raises the question of why outcomes differed between patients whose samples were or were not sent for central testing. Unfortunately, as central testing was optional, reasons for not testing centrally were not collected prospectively and it is difficult to speculate on explanations for these surprising findings from post hoc exploratory analyses.

A limitation of the study is that the enrolled patient population included very few patients outside the more selected population enrolled in IMpassion130, and therefore we were unable to gain meaningful insight into the safety and efficacy of atezolizumab plus nab-paclitaxel in patients with ECOG PS 2, pre-existing autoimmune conditions, CNS metastases or prior anti-PD-(L)1 therapy. It was not foreseen to collect the reasons for not enrolling patients into the trial. Possible explanations for the small numbers of patients in broader populations of specific interest include the absence of such patients in everyday practice at the participating centres, physician bias or concerns about safety in more frail patients, patient reluctance to participate and/or competing clinical trials, but we have no evidence to support or refute these hypotheses. As with other recent phase III trials in the first-line aTNBC setting, none of the patients had previously received anti-PD-(L)1 therapy, but pembrolizumab-containing neoadjuvant therapy is now considered the standard of care for patients with higher risk early TNBC [20,21]. Information on *BRCA* mutation status was not collected prospectively. Furthermore, although it was not standard during the period of study conduct, it would have been interesting to collect and explore the EL1SSAR dataset according to HER2-low and HER2-ultralow status.

At the other end of the spectrum, continuation of atezolizumab and/or nab-paclitaxel treatment for >3 years in 7% of patients indicates a very tolerable regimen but raises the question of optimal treatment duration. It remains unknown whether immunotherapy should be continued until disease progression or unacceptable toxicity or stopped after a fixed duration in patients with sustained disease control. The good tolerability of atezolizumab-containing therapy also supports the potential to combine anti-PD-(L)1 therapy with antibody–drug conjugates, a strategy that has already been tested in the BEGONIA (durvalumab and datopotamab deruxtecan, irrespective of PD-L1 status [22]) and MORPHEUS (atezolizumab and sacituzumab govitecan [23]) studies, and most recently has shown benefit in the ASCENT-04/KEYNOTE-D19 phase III trial (pembrolizumab and sacituzumab govitecan versus pembrolizumab and chemotherapy in PD-L1-positive aTNBC) [18].

In conclusion, despite the lack of evidence generated in understudied populations, the EL1SSAR trial suggests that populations selected by local PD-L1 testing have similar outcomes to patients enrolled in the pivotal IMpassion130 trial. Local PD-L1 testing appeared to provide a relevant selection tool for the proposed treatment scheme. The implications of testing disparities will become more important with the advent of antibody–drug conjugates, which offer effective first-line combination regimens, including OS benefit over existing chemoimmunotherapy regimens, for patients with PD-L1-positive aTNBC [18].

## Disclosures

LG reports consulting fees from Revolution Medicines, Zymeworks, Roche, Menarini Ricerche and Denali Therapeutics Inc, participation on data safety monitoring or advisory boards for AstraZeneca, Roche, Synaffix, Zymeworks, Daiichi Sankyo, BeiGene and Oncolytics Biotech, coinventor of “European patent application n. 12195182.6 and 12196177.5 entitled PD-L1 expression in anti-HER2 therapy” (Roche, no compensation provided) and Chair of the International Breast Cancer Research Committee Fondazione Michelangelo. SD reports participation on advisory/scientific boards (all honoraria to institution) for Elsan, Gilead and Novartis, membership on steering committees (all honoraria to institution) for BMS, Roche/Genentech and Sanofi, and funding from Banque des Territoires/France 2030 and the European Commission. Given her role as Editorial Board Member, SD had no involvement in the peer review of this article and has no access to information regarding its peer review. EC reports honoraria for advisory board participation from AstraZeneca, Avenzo, BeiGene, Daiichi Sankyo, Lilly, MSD, Novartis, Pfizer, Reveal Genomics and Roche, invited speaker honoraria from Gilead, Lilly, Pfizer and Roche, speakers' bureau for Roche, research grants (to institution) from Daiichi Sankyo, Roche and Seagen and travel/accommodation support from AstraZeneca. OT reports speaker honoraria from Roche, Pfizer, Novartis–Sandoz, Lilly, MSD, AstraZeneca, Pierre Fabre, Seagen, Daiichi Sankyo, Gilead, Eisai, Menarini-Stemline, Veracyte, Viatris and Exact Sciences, support and/or travel for attending meetings from Roche, Pfizer, Novartis–Sandoz, Lilly, MSD, AstraZeneca, Seagen, Daiichi Sankyo and Gilead and participation in data safety monitoring or advisory boards for Roche, Pfizer, Novartis–Sandoz, Lilly, MSD, AstraZeneca, Seagen and Daiichi Sankyo. CC reports invited speaker honoraria from AstraZeneca and Novartis. MHA reports fees for invited speaker engagements from Novartis, GSK, MSD, Pfizer, AstraZeneca, Lilly and Daiichi Sankyo and support (fees and travel) for international meeting participation from PharmaMar, Daiichi Sankyo, AstraZeneca, Lilly, MSD and Roche. IP reports participation on advisory/scientific committees for Gilead, Lilly, Novartis, Roche/Genentech, Pfizer, MSD, Gentili, Genetic, AstraZeneca and Daiichi Sankyo and travel grants from Roche, MSD and AstraZeneca. MC reports a leadership role (non-financial interest) as the International Breast Cancer Study Group Scientific Committee Co-Chair. NA reports meeting/travel support from Pfizer. VG reports consulting fees for advisory board participation from BMS, Pierre Fabre, Regeneron and Daiichi Sankyo, speaker honoraria from Novartis and meeting/travel support from MSD, GenFleet Therapeutics and Pierre Fabre. SB reports honoraria for advisory board participation from AstraZeneca, Eli Lilly, Gilead, Lek, MSD, Novartis, Pfizer and Roche, uncompensated roles as local principal investigator for trials by Novartis and Roche, and leadership of the Slovenian Senologic Society. IB reports honoraria for advisory boards from Adelphi Targis, AstraZeneca, Bristol-Myers Squibb, Celgene, Cinfa, Daiichi Sankyo, Eisai, Gilead, Grünenthal, GSK, Lilly, MSD, Novartis, Pfizer, Pierre Fabre, Roche and Seagen, speaker honoraria from Pfizer, TACTICS and Transworld Editors, honoraria for medical monitor role for Medica Scientia Innovation Research (MEDSIR) and research funding (to institution) from Agendia, AstraZeneca, Lilly, Pfizer and Roche. CA-S reports speaker honoraria from AstraZeneca, Novartis, Roche, Eisai, Gilead, MSD and Eli Lilly. PSM reports honoraria for speaker engagements from Novartis, meeting/travel support from AstraZeneca, Gilead, Pfizer, Lilly, MSD, Novartis and Roche, participation on advisory or data safety monitoring boards for Novartis and (unpaid) board membership of the Sociedade Portuguesa de Oncologia. YGL, CY and KK report employment with Genentech and stock ownership of Roche. JM reports employment and stock ownership for F. Hoffmann-La Roche Ltd, Basel, Switzerland. MM reports honoraria from AstraZeneca, Lilly, Amgen, Roche/Genentech, Novartis and Pfizer, advisory roles for AstraZeneca, Amgen, Taiho Oncology, Roche/Genentech, Novartis, PharmaMar, Eli Lilly, Puma Biotechnology, Taiho Oncology, Daiichi Sankyo and Pfizer, speakers’ bureau for Lilly/ImClone, Genentech/Roche and Pierre Fabre, and research funding from Novartis, Roche and Puma Biotechnology. The remaining authors declare no conflict of interest beyond medical writing support (funded by F. Hoffmann-La Roche Ltd) for this manuscript.

## Data sharing

Qualified researchers may request access to individual patient-level data through the clinical study data request platform (https://vivli.org/). Further details on Roche's criteria for eligible studies are available here (https://vivli.org/members/ourmembers/). For further details on Roche's Global Policy on the Sharing of Clinical Information and how to request access to related clinical study documents, see here (https://www.roche.com/research_and_development/who_we_are_how_we_work/clinical_trials/our_commitment_to_data_sharing.htm).

## Funding

This work was supported by 10.13039/100021981F. Hoffmann-La Roche Ltd (no grant number), which was involved in designing the study and collecting, analysing and interpreting the data. Employees of the sponsor are among the co-authors and F. Hoffmann-La 10.13039/100004337Roche Ltd funded third-party medical writing support for this manuscript, provided by Jennifer Kelly (Medi-Kelsey Ltd, Ashbourne, 10.13039/100007472UK). The decision to submit the manuscript was the responsibility of the first author.

## CRediT authorship contribution statement

**L. Gianni:** Conceptualization, Investigation, Methodology, Resources, Supervision, Writing – original draft, Writing – review & editing. **S. Delaloge:** Conceptualization, Investigation, Methodology, Resources, Writing – original draft, Writing – review & editing. **E. Ciruelos:** Conceptualization, Investigation, Methodology, Resources, Writing – review & editing. **O. Trédan:** Conceptualization, Investigation, Methodology, Resources, Writing – review & editing. **C. Castañeda:** Investigation, Resources, Writing – review & editing. **M.H. Abreu:** Investigation, Resources, Writing – review & editing. **I. Paris:** Investigation, Resources, Writing – review & editing. **M. Colleoni:** Investigation, Resources, Writing – review & editing. **G. Scandurra:** Investigation, Resources, Writing – review & editing. **M. Kwiatkowski:** Investigation, Resources, Writing – review & editing. **N. Antone:** Investigation, Resources, Writing – review & editing. **V. Gregorc:** Investigation, Resources, Writing – review & editing. **P. Tagliaferri:** Investigation, Resources, Writing – review & editing. **S. Borštnar:** Investigation, Resources, Writing – review & editing. **I. Blancas:** Investigation, Resources, Writing – review & editing. **M. Raimondo:** Investigation, Resources, Writing – review & editing. **M. Alfie:** Investigation, Resources, Writing – review & editing. **C. Arce-Salinas:** Investigation, Resources, Writing – review & editing. **P.S. Madeira:** Investigation, Resources, Writing – review & editing. **Y.G. Lin:** Writing – review & editing. **C. Ye:** Data curation, Formal analysis, Software, Validation, Visualization, Writing – original draft, Writing – review & editing. **K. Koli:** Writing – review & editing. **J. Mouta:** Conceptualization, Data curation, Methodology, Project administration, Supervision, Writing – original draft, Writing – review & editing. **M. Martin:** Conceptualization, Investigation, Methodology, Resources, Supervision, Writing – review & editing.

## Figures and Tables

**Table:** 

Number of patients with ≥1 AE	Event rate (95% CI)
1	0.56% (0.01–3.06%)
2	1.11% (0.13–3.96%)
3	1.67% (0.35–4.79%)
4	2.22% (0.61–5.59%)
5	2.78% (0.91–6.36%)
6	3.33% (1.23–7.11%)
7	3.89% (1.58–7.85%)
8	4.44% (1.94–8.57%)
9	5.00% (2.31–9.28%)
10	5.56% (2.70–9.98%)
11	6.11% (3.09–10.67%)
12	6.67% (3.49–11.36%)
13	7.22% (3.90–12.03%)
14	7.78% (4.32–12.71%)
15	8.33% (4.74–13.37%)

AE = adverse event; CI = confidence interval.

Based on *n* = 180, an AE with a true rate of 1% has an 84% probability of being observed in the study, an AE with a true rate of 0.5% has a 59% probability of being observed and an AE with a true rate of 0.1% has a 16% probability of being observed.

## GLOSSARY

AE: adverse event
AESI: adverse event of special interest
ALT: alanine aminotransferase
AST: aspartate aminotransferase
aTNBC: advanced triple-negative breast cancer
CI: confidence interval
CNS: central nervous system
ECOG PS: Eastern Cooperative Oncology Group performance status
eTNBC: early triple-negative breast cancer
NA: not applicable
NE: not estimable
OS: overall survival
PFS: progression-free survival
RECIST: Response Evaluation Criteria in Solid Tumours
SAE: serious adverse event
TNBC: triple-negative breast cancer
WBC: white blood cell

## Appendix A Pre-existing autoimmune diseases and immune deficiencies

Patients with a history of autoimmune disease were allowed to enrol if the condition had been controlled on stable treatment (i.e., same treatment, same dose) for the preceding 12 weeks, with the following exceptions:

1. Concurrent abatacept or belatacept treatment, unless therapy had been withdrawn for >8 weeks
2. A history of serious or life-threatening immune-mediated events
3. More than one concomitant autoimmune disease at the time of study entry, unless one of them was:
  i Autoimmune-mediated hypothyroidism on a stable dose of thyroid replacement hormone
  ii) Controlled type I diabetes mellitus on a stable dose of insulin regimen
  iii) A medical history of such entities as atopic disease or childhood arthralgias, where the clinical suspicion of autoimmune disease was low. In addition, transient autoimmune manifestations of an acute infectious disease that resolved upon treatment of the infectious agent were not excluded (e.g., acute Lyme arthritis).

Investigators were to use caution when considering atezolizumab for patients who had previously experienced a severe or life-threatening skin adverse reaction or pericardial disorder while receiving another immunostimulatory anti-cancer agent.

Patients with autoimmune disease were ineligible if they had received treatment with systemic corticosteroids or other systemic immunosuppressive medications (including but not limited to prednisone, dexamethasone, cyclophosphamide, azathioprine, methotrexate, thalidomide and anti-tumour necrosis factor agents) within 2 weeks before the first dose of study treatment (Cycle 1, Day 1), or were anticipated to require systemic immunosuppressive medications during the study. Acute, low-dose systemic immunosuppressant medications (e.g., a one-time dose of dexamethasone for nausea) were permitted. The use of inhaled corticosteroids for chronic obstructive pulmonary disease, mineralocorticoids (e.g., fludrocortisone) for patients with orthostatic hypotension and low-dose supplemental corticosteroids for adrenocortical insufficiency was allowed.

## Appendix C Participating investigators

MA Colleoni, I Paris, G Scandurra, P Tagliaferri, V Gregorc, C Gridelli, L Biganzoli, G Bianchini, D Sartori, A Pazzola, G Rizzo, L Livi, A Ferro, ME Cazzaniga, A Fontana, R Berardi, M Montedoro (Italy), MI Blancas Lopez-Barajas, A Rodriguez Lescure, S Morales Murillo, E Ciruelos Gil, F Ayala De La Peaña, E Dalmau Portulas, E Galve Calvo, A Godoy Ortiz, C Rodriguez Sanchez, E Alvarez Gomez, I Fernandez Perez, Y Fernandez Perez (Spain), M Henriques Abreu, P Madeira, S Braga, M Brito, A Martins, H Mansinho (Portugal), M Kwiatkowski, A Mruk, R Surma-Wlodarczyk, D Sawka (Poland), M Schenker, N Antone, A Ungureanu (Romania), C Castañeda, CL Acevedo Zanabria (Peru), O Trédan, E Legouffe, V Simal Lorgis, I Iurisci, S Delaloge, J Grosjean (France), M Biro, A Uhlyarik, Z Horvath, K Mahr, P Arkosy (Hungary), M Alfie, M Raimondo, F Perazzo (Argentina), S Borstnar, M Ravnik (Slovenia), CH Arce Salinas, RC Grajales Alvarez (Mexico), P Holeckova, J Pribylova, D Vrana (Czech Republic), C Sanchez, P Rodriguez (Chile).

## Footnotes

Appendix D Supplementary data to this article can be found online at https://doi.org/10.1016/j.breast.2026.104836.
