# Supplementary material for: Primary results from EL1SSAR, a prospective phase IIIb study of first-line atezolizumab plus nab-paclitaxel therapy for patients with PD-L1-positive advanced triple-negative breast cancer
Source: Breast. 2026 Jun 8;88:104836. doi: 10.1016/j.breast.2026.104836 (PMC13315958; doi:10.1016/j.breast.2026.104836)
Supplement: Multimedia component 1 [file mmc1.docx]

**Primary results from EL1SSAR, a prospective phase IIIb study of first-line atezolizumab plus nab-paclitaxel therapy for patients with PD-L1-positive advanced triple-negative breast cancer**

**L. Gianni et al.**

**Supplementary materials**

| **Supplementary** **Table A.1. Details of grade ≥2 immune-mediated AEs (*n* = 182).** Immune-mediated AEs are defined as events that resemble autoimmune diseases, and are known side effects of immune checkpoint inhibitors, including atezolizumab. Qualifying events are those AEs of special interest that were ongoing upon the initiation of systemic corticosteroid therapy and where the systemic corticosteroid therapy was administered no later than 30 days from the start of the AE. | | |
| --- | --- | --- |
| **AE** | **No. of patients (%)** | **Grade (onset day)** |
| Hepatitis (laboratory abnormality) | 8 (4.4) |  |
| ALT increased | 4 (2.2) | G3 (d22)^a^, G2 (d37), G3 (d60)^b^, G2 (d433) |
| AST increased | 4 (2.2) | G2 (d22)^a^, G3 (d29), G3 (d60)^b^, G3 (d129)^c^ |
| Transaminases increased | 2 (1.1) | G4 (d43)^d^, G3 (d67) |
| Blood bilirubin increased | 1 (0.5) | G3 (d71)^d^ |
| Gamma-glutamyl transferase increased | 1 (0.5) | G3 (d129)^c^ |
| Hypertransaminasaemia | 1 (0.5) | G2 (d22)^a^ |
| Hepatitis (diagnosis) | 4 (2.2) |  |
| Hepatic cytolysis | 1 (0.5) | G3 (d27) |
| Hepatic failure | 1 (0.5) | G3 (d287) |
| Hepatitis | 1 (0.5) | G3 (d260)^c^ |
| Immune-mediated hepatitis | 1 (0.5) | G3 (d28) |
| Pneumonitis | 3 (1.6) |  |
| Pneumonitis | 3 (1.6) | G3 (d25), G5 (d109),^e^ G3 (d472)^f^ |
| Acute interstitial pneumonitis | 1 (0.5) | G3 (d621)^f^ |
| Colitis | 2 (1.1) | G2 (d238), G2 (d387) |
| Rash | 2 (1.1) |  |
| Rash maculopapular | 1 (0.5) | G3 (d70) |
| Rash pustular | 1 (0.5) | G2 (d108, d139, d192) |
| Hypothyroidism | 1 (0.5) | G2 (d60) |
| Myelitis | 1 (0.5) | G3 (d44) |
| Myocarditis | 1 (0.5) | G2 (d43) |
| Myositis | 1 (0.5) | G2 (d570) |
| Nephritis | 1 (0.5) | G3 (d42) |
| Ocular inflammatory toxicity | 1 (0.5) |  |
| Chorioretinopathy | 1 (0.5) | Worsening to G2 (d57) |

AE, adverse event; AESI, adverse event of special interest; ALT, alanine aminotransferase; AST, aspartate aminotransferase; d, day; G, grade.

AEs encoded using MedDRA version 27.1. AESIs use Medical Concept Groupings version 6.17. Multiple occurrences of the same preferred term in an individual patient were counted only once.

^a^G3 ALT increased, G2 AST increased and G2 hypertransaminasaemia in the same patient, all on day 22.

^b^G3 ALT increased and G2 AST increased in the same patient, both on day 60.

^c^G3 AST increased and G3 gamma-glutamyl transferase increased in the same patient, both on day 129, followed by G3 hepatitis on day 260.

^d^G4 transaminases increased (day 43) and G3 blood bilirubin increased (day 71) in the same patient.

^e^G5 pneumonitis considered by the treating physician to be caused by fungal infection.

^f^G3 pneumonitis (day 472) and G3 acute interstitial pneumonitis (day 621) in the same patient.

| **Supplementary Table A.2. Baseline characteristics of the exploratory subgroup with PFS >2 years (*n =* 27)** | |
| --- | --- |
| **Characteristic** | **No. of patients (%)** |
| Age, years |  |
| Median (range) | 52 (37–82) |
| 18–40 | 2 (7) |
| 41–64 | 19 (70) |
| ≥65 | 6 (22) |
| Sex |  |
| Female | 27 (100) |
| Race |  |
| American Indian/Alaska Native | 3 (11) |
| Asian | 0 |
| Black/African American | 0 |
| White | 22 (81) |
| Unknown | 2 (7) |
| ECOG PS |  |
| 0 | 21 (78) |
| 1 | 6 (22) |
| Histology^a^ |  |
| Invasive breast cancer of no special type | 20 (74) |
| Medullary | 1 (4) |
| Tubular | 1 (4) |
| Other | 2 (7) |
| Not otherwise specified | 5 (19) |
| Grade |  |
| Well differentiated | 2 (7) |
| Moderately differentiated | 3 (11) |
| Poorly differentiated | 14 (52) |
| Unknown | 8 (30) |
| Disease status |  |
| Locally advanced unresectable | 6 (22) |
| Metastatic | 21 (78) |
| De novo metastatic | 9 (33) |
| Metastatic sites |  |
| Liver | 2 (7) |
| Lung | 10 (37) |
| Lymph node status |  |
| Positive | 9 (33) |
| Negative | 10 (37) |
| Positive | 8 (30) |

ECOG PS, Eastern Cooperative Oncology Group performance status; PFS, progression-free survival.

^a^More than 1 answer possible.

| **Supplementary** **Table A.3. Summary of outcomes in pre-specified subgroups** | | | | | | | |
| --- | --- | --- | --- | --- | --- | --- | --- |
|  |  | **Patients with AEs, *n* (%) [95% CI]** | | | | **Median, months (95% CI)** | |
| **Population** | **N** | **Grade ≥3 AEs** | **Grade ≥2 immune-mediated AEs** | **AEs** | **SAEs** | **PFS** | **OS** |
| All patients | 182 | 85 (47) [39–54] | 22 (12) [8–18] | 174 (96) [92–98] | 30 (16) [11–23] | 7.4 (5.6–10.6) | 27.0 (22.0–33.8) |
| Centrally confirmed PD-L1+ | 66 |  |  |  |  | 11.1 (7.4–16.8) | NE (29.4–NE) |
| CNS metastases | 5 | 3 (60) [15–95] | 1 (20) [1–72] | 5 (100) [48–100] | 0 (0) [0–52] | 3.6 (2.9–NE) | 6.3 (4.7–NE) |
| ECOG PS ≥2 | 3 | 3 (100) [29–100] | 1 (33) [1–91] | 3 (100) [29–100] | 1 (33) [1–91] | 0.9 (0.3–NE) | 0.9 (0.3–NE) |
| No prior anti-cancer therapy | 76 | 36 (47) [36–59] | 13 (17) [9–27] | 73 (96) [89–99] | 15 (20) [11–30] | 8.7 (5.3–12.2) | 33.8 (27.1–NE) |
| Prior anti-cancer therapy for eTNBC | 106 | 49 (46) [36–56] | 9 (8) [4–16] | 101 (95) [89–98] | 15 (14) [8–22] | 7.2 (5.4–10.8) | 22.3 (18.3–27.2) |
| Taxane | 92 | 44 (48) [37–59] | 8 (9) [4–16] | 89 (97) [91–99] | 14 (15) [9–24] | 7.2 (5.3–10.2) | 22.3 (17.5–27.2) |
| Non-taxane | 14 | 5 (36) [13–65] | 1 (7) [0–34] | 12 (86) [57–98] | 1 (7) [0–34] | 11.1 (4.4–14.1) | 20.7 (15.9–NE) |

AE, adverse event; CI, confidence interval; CNS, central nervous system; ECOG PS, Eastern Cooperative Oncology Group performance status; eTNBC, early triple-negative breast cancer; NE, not estimable; OS, overall survival; PFS, progression-free survival; SAE, serious adverse event.

| **Supplementary** **Table A.4. PD-L1 testing concordance (*n* = 97)** | | | |
| --- | --- | --- | --- |
| **Central testing** | **Local testing** | | **Total** |
|  | **PD-L1+** | **PD-L1–** |  |
| **PD-L1+** | 65 | 1^a^ | 66 |
| **PD-L1–** | 31 | 0 | 31 |
| **Total** | 96 | 1 | 97 |

^a^PD-L1 recorded as 0 per local assessment.

| **Supplementary** **Table A.5. Most common subsequent anti-cancer therapies (post hoc analysis) (*n =* 182)** | |
| --- | --- |
| **Anti-cancer therapy in follow-up** | **No. of patients (%)** |
| Any | 109 (60) |
| Platinum | 60 (33) |
| Capecitabine | 57 (31) |
| Gemcitabine | 41 (23) |
| Antibody–drug conjugate | 32 (18) |
| Anthracycline (± cyclophosphamide) | 27 (15) |
| Eribulin | 23 (13) |
| Taxane | 19 (10) |
| Vinorelbine | 14 (8) |
| PD-(L)1 inhibitor | 10 (5) |
| PARP inhibitor | 7 (4) |
| Other cyclophosphamide-containing regimen | 5 (3) |
